# A Novel Manufacturing Process for Compact, Low-Weight and Flexible Ultra-Wideband Cavity Backed Textile Antennas

**DOI:** 10.3390/ma11010067

**Published:** 2018-01-03

**Authors:** Dries Van Baelen, Sam Lemey, Jo Verhaevert, Hendrik Rogier

**Affiliations:** Department of Information Technology, Ghent University/imec, Technologiepark-Zwijnaarde 15, 9052 Ghent, Belgium; Sam.Lemey@UGent.be (S.L.); Jo.Verhaevert@UGent.be (J.V.); Hendrik.Rogier@UGent.be (H.R.)

**Keywords:** wearable antennas, ultra-wideband (UWB), textile antennas, substrate integrated waveguide (SIW), Internet of Things (IoT), flexible electronics, body centric communication

## Abstract

A novel manufacturing procedure for the fabrication of ultra-wideband cavity-backed substrate integrated waveguide antennas on textile substrates is proposed. The antenna cavity is constructed using a single laser-cut electrotextile patch, which is folded around the substrate. Electrotextile slabs protruding from the laser-cut patch are then vertically folded and glued to form the antenna cavity instead of rigid metal tubelets to implement the vertical cavity walls. This approach drastically improves mechanical flexibility, decreases the antenna weight to slightly more than 1 g and significantly reduces alignment errors. As a proof of concept, a cavity-backed substrate integrated waveguide antenna is designed and realized for ultra-wideband operation in the [5.15–5.85] GHz band. Antenna performance is validated in free space as well as in two on body measurement scenarios. Furthermore, the antenna’s figures of merit are characterized when the prototype is bent at different curvature radii, as commonly encountered during deployment on the human body. Also the effect of humidity content on antenna performance is studied. In all scenarios, the realized antenna covers the entire operating frequency band, meanwhile retaining a stable radiation pattern with a broadside gain above 5 dBi, and a radiation efficiency of at least 70%.

## 1. Introduction

In the most recent years, numerous applications have arisen that would benefit greatly from Smart Fabrics and Interactive Textiles (SFIT) technologies. These applications include, among others, on-body communication nodes for first responders [1,2], medical implant communication infrastructure [3,4], personal locator beacons implemented in life jackets [5,6] and Internet of Things (IoT)-applications [7,8,9]. To achieve a deep market penetration of such systems, consumer requirements concerning wearability need to be satisfied. This can be achieved through textilization of the aforementioned components.

Up to now, some textile systems have been demonstrated that address a number of the issues arising with wearable communication systems. In [10], the first full-textile substrate integrated waveguide (SIW) antenna based on a conductive coated fabric and metal long sail eyelets is proposed. The antenna exhibits low coupling to the human body, yet long sail eyelet punching requires contact pressure to create good contact between different electrotextile layers, which causes compression of the foam substrate. Furthermore, this antenna employs metal long sail eyelets as vertical walls of the SIW cavity, resulting in a heavier and larger antenna structure [11]. The work in [8] addresses some of these issues, as one-piece tube eyelets, henceforth called “tubelets”, are used [8,9,12] instead of two-part long sail eyelets used in [10], thereby removing the concern of compression due to the additional part of the latter. Furthermore, the use of two resonant modes results in a broader bandwidth. In [12], both metal tubelets and conductive coated fabrics are used, offering the same advantages as in [8]. This antenna topology provides a very high degree of miniaturization, and exhibits two modes. Thereby, it demonstrates the effectiveness of the antenna at higher frequencies. All fabrication methods until now require two electrotextile layers, an antenna substrate layer and a significant amount of tubelets to implement the antenna cavity. This results in an elaborate manufacturing procedure with a high number of fabrication steps, as, first, three layers of material need to be patterned; next, judicious alignment between all layers should be guaranteed; and, finally, multiple rows of tubelets need to be inserted at the correct positions. The work in [13] also implements the cavity top and bottom planes by a conductive coated fabric, but utilizes embroidery to construct the vertical cavity walls. This results in a fully textilized design that is more efficient than antennas where the cavity top and bottom planes are realized by embroidery [14]. Furthermore, as the thin electrotextile threads substitute the vias, the antenna size is reduced even further. However, the vertical wall conductivity is low and rather inaccurate, which impedes reproducibility, and special attention has to be paid to the stitch direction with respect to current flow [15].

This paper proposes a novel production method to implement SIW technology in textile materials. Now, the antenna cavity is constructed out of a single laser-cut electrotextile layer that is folded around the antenna substrate, thereby implementing all cavity walls without requiring additional tubelet punching or embroidery steps and the corresponding alignment issues. As a result, the fabrication procedure requires fewer manufacturing steps, and yields highly efficient, fully textilized antennas that are not only easier to design, but are also more conveniently fabricated at higher accuracy. Furthermore, these antennas are lightweight, more compact and mechanically more flexible as a result of substituting the heavy metal tubelets by electrotextile slabs. Since ripping of the electrotextile around the metal tubelets has now been avoided, the robustness of the antenna increases. Even further miniaturization is obtained by using a Hirose ultra-small surface mount coaxial connector (U.FL connector) [16] instead of a rather bulky SubMiniature version A (SMA) connector [9,10,17].

A representative design is discussed to demonstrate the novel fabrication process. Therefore, an antenna for operation in the [5.15–5.85] GHz band has been developed and fabricated, based on the hybrid cavity mode operation principle introduced in [18]. The antenna covers the entire frequency band, and maintains a stable radiation pattern with a broadside gain above 5 dBi and a radiation efficiency of at least 70%, measured both in free space and realistic on body conditions.

This paper is organized as follows. In Section 2, the antenna topology and antenna operation principle are discussed. Specific attention is paid to the choice of materials. In Section 3, the manufacturing process is elaborated. Four essential fabrication steps are described to construct the antenna. Section 4 shows the influence of essential design parameters on the behavior of the antenna and thus motivates the chosen antenna parameter values. Measurements were performed both in free space and realistic on-body operating conditions. These are discussed in Section 5.

## 2. Antenna Design and Material Selection

In this section, the antenna design principle and the figures of merit to which it has to comply are discussed. Furthermore, an insight is given in the utilized antenna materials.

### 2.1. Antenna Design

#### 2.1.1. Design Goals

The antenna will be designed for operation in all unlicensed national information infrastructure (UNII) radio bands. As such, ultra-wideband (UWB) antenna operation is required with an impedance bandwidth extending from 5.15 GHz to 5.85 GHz. This means that the magnitude of the reflection coefficient $|S_{1,1}|$ should remain below −10 dB and an antenna efficiency of at least 70% should be achieved over the entire band, while maintaining stable radiation characteristics. The first requirement will guarantee that at most 10% of the maximally available power is reflected back by the antenna and, therefore, 90% of that power is injected into the antenna. In the presence of losses, this power will either be radiated or dissipated into heat. A radiation efficiency of at least 70% will ensure that maximally 30% of the power injected into the antenna is transferred into heat, while minimally 70% is radiated. Meeting these requirements in the considered frequency band of operation allows, for example, to set up wireless communication using the 802.11 ac protocol. From the end user’s perspective, the antenna system needs to be highly flexible, robust (and thus also resistant against crumpling), lightweight, and invisibly integrated. From the system engineer’s perspective, this means that the radiation characteristics of the antenna need to be stable when the antenna is subject to bending over different curvature radii, an effect that is commonly encountered when the antenna is worn on the human body. Furthermore, when deploying an antenna on the human body, part of the radiated power will be absorbed and transformed into heat in the body itself, in addition to the power dissipated in the antenna itself. Hence, the antenna’s radiation efficiency will typically decrease when the antenna is placed on the human body. Therefore, we require that the antenna radiates its energy mainly within half a hemisphere pointing away from the body, such that power absorbed by the human body is minimized. In doing so, the decrease in antenna efficiency by deploying the antenna on the human body will be minimized and a high and stable radiation efficiency can be obtained, which is of high importance given the limited available power in on-body applications. For the end user’s safety, such an antenna topology that exhibits low coupling to the human body will also reduce the wearer’s radiofrequency-field exposure. The front-to-back ratio of the antenna is a very suitable figure of merit to indicate the isolation between the human body and the antenna, as it describes the amount of power radiated away from the human body with respect to the power radiated towards the human body. This ratio is characterized in free space conditions, since the power radiated towards the body cannot be measured in actual deployment conditions, because this power is directly absorbed by the body. Therefore, in free-space conditions, we require a front-to-back ratio of at least 6 dB, meaning that the antenna radiates four times more power away from the body than towards the body. In this respect, one typically defines the antenna’s directivity as the power radiated by the antenna along a certain direction over the power radiated by an isotropic antenna, in which the same amount of power is injected. In contrast to the directivity, the antenna gain also takes losses in the antenna into account. The antenna gain is found by multiplying the directivity by the antenna’s radiation efficiency.

#### 2.1.2. Antenna Topology

**Figure 1:**
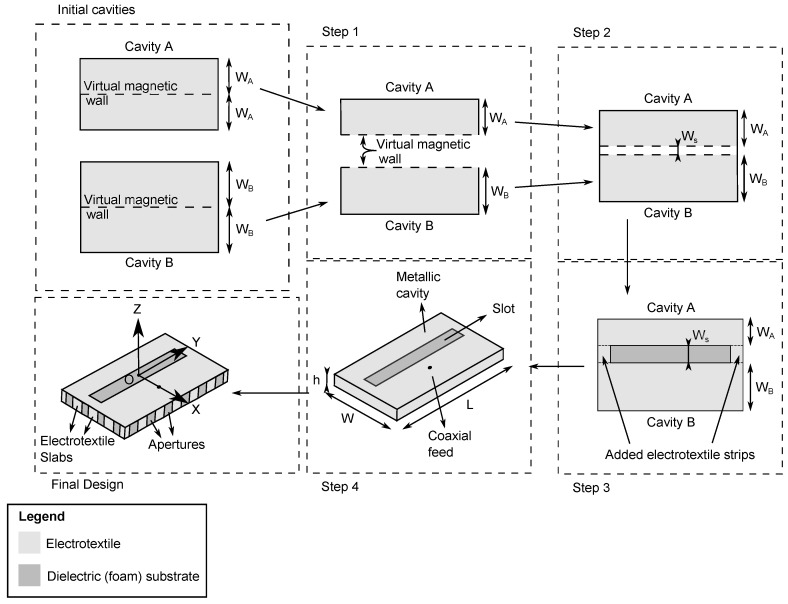
Coupled half-mode cavity design steps.

In order to fulfill the design goals, a cavity-backed slot antenna topology is chosen. Such antennas are composed of a metallic cavity, filled with a dielectric material, in which a slot is cut out, as seen in Figure 1. The metal walls ensure that the electromagnetic waves only radiate through this slot, being in the hemisphere along the positive Z-axis. Therefore, if the human body is located within the opposite hemisphere, along negative Z values, it will be shielded from radiation, thereby fulfilling the radiation exposure constraint. To achieve mechanical flexibility, a closed-cell expanded-rubber foam is chosen as dielectric substrate, whereas the metal walls of the cavity are implemented in a conducting electrotextile. However, radiating cavities are typically very narrowband, as only one resonant cavity mode is excited [19]. To remedy this, the cavity bandwidth enhancement technique proposed in [18] is applied, providing a −10 dB impedance bandwidth that covers the entire desired frequency band, as explained below.

#### 2.1.3. Operation Principle

**Figure 2:**
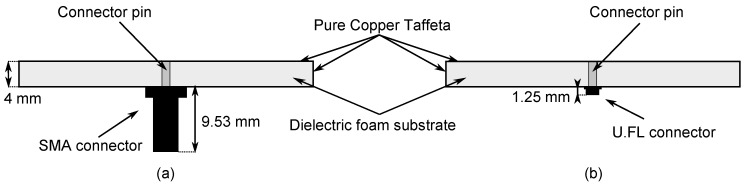
Cross-section of the textile ultra-wideband SIW antenna with SMA connector (**a**) and U.FL connector (**b**).

Figure 1 schematically illustrates the adopted bandwidth enhancement technique, where two half-mode cavities are brought together to achieve ultra-wideband behavior: Since the field distribution of the $TE_{110}$ mode inside a rectangular cavity is symmetrical, the horizontal symmetry plane of the cavity can be considered as a virtual magnetic wall (Figure 1 step 1) [18]. If now two differently dimensioned half-mode cavities are placed in close proximity within a single antenna footprint, they exhibit a strong coupling to each other. This strong coupling causes mode bifurcation [20], yielding two distinct resonances at frequencies that are controlled by the dimensions of each subcavity and their respective spacing $W_{s}$ (step 2). Since both resonance frequencies are reasonably close to each other, thorough computer-aided optimization of the aforementioned resonance frequencies allows the coupled half-mode cavities to cover the entire frequency band of operation. At both sides of the radiating slot, electrotextile strips are added to keep both half-mode cavities together, as shown in step 3. These strips will also be used to further tune coupling between both half-mode cavities. In step 4 on Figure 1, the cavity is excited by a coaxial probe feed. Therefore, as shown in Figure 2, the inner and outer conductor of the coaxial feed are connected to the antenna slot plane and back plane, respectively. Well-chosen and optimized positioning of the coaxial feed ensures that a loop is made, which creates a magnetic field inside the cavity that couples to the magnetic fields of both subcavity modes. As such, the E-field of the antenna element lies within the XZ-plane, and the H-field lies within the YZ-plane. This results in an antenna that is linearly polarized along the X-axis. Meanwhile, it has to be kept in mind that mechanical bending causes a solid electrotextile wall to crumple. This undesirable effect is avoided by implementing vertical walls of the cavity as a series of closely spaced electrotextile slabs, instead of solid metal vertical walls commonly used in cavities. This results in the final flexible cavity-backed slot antenna design.

### 2.2. Antenna Materials

As a dielectric foam substrate, a closed-cell expanded rubber, commonly used as a protective foam in firefighter jackets, is chosen and placed in the antenna as shown in Figure 1 and Figure 2 [21]. This substrate is flexible, fire-resistant, water-repellent, recovers easily from compression and has a thickness of 4 mm, which is sufficient to achieve the desired bandwidth based on the proposed antenna topology. As a conductive textile, a copper-plated Pure Copper Taffeta electrotextile with a surface resistivity of 0.05 Ω/□ is used [8,22]. A low surface resistivity is necessary to reduce conductive losses in the cavity, and thus contributes to a high antenna efficiency [13]. The electrotextile is glued to the substrate using a thermally-activated adhesive sheet that attaches to the coated fabric, as used in [8].

As displayed in Figure 2, two different types of connectors are investigated to implement the coaxial feed probe. SMA connectors are commonly used in textile designs [8,9,13], and have the immediate advantage that they already possess a pin, which will serve as a feed via to generate the excitation in the cavity [10], as is explained earlier. Yet, as shown in Figure 2, SMA connectors are rather bulky compared to the rest of the design. Because of the importance of miniaturization in wearable electronics, the small size and lightweight properties of U.FL connectors justify investigating the use of this connector type on the antenna. Choosing a U.FL connector over an SMA connector decreases the antenna mass from 3.3 g to only 1.3 g, implying a weight reduction by 60%. However, in that case, a connector pin needs to be soldered to the U.FL connector first. For this, a 1-mm-diameter brass-gold pin is chosen.

## 3. Manufacturing Process

The manufacturing process mainly consists of four steps, further explained below. First, the electrotextile is vacuum laminated to the adhesive sheet, after which both are laser cut. The substrate is laser cut as well, albeit in a different form. Then, a judicious alignment procedure is used to attach and wrap the electrotextile-adhesive patch around the substrate. In a final step, a connector with feed pin is inserted into the cavity.

### 3.1. Vacuum Lamination

To ensure that the electrotextile attaches to the substrate in a uniform way, a thermally-activated adhesive sheet has been used. To guarantee uniform adhesion to both the substrate and the electrotextile without glue absorption in either the electrotextile or the substrate, controlled process conditions are required. A Hotronix^®^ (Stahls, Carmichaels, PA 15320, USA, online: https://www.hotronix.com) Air Fusion heat press is applied to thermally activate the glue with a homogeneously applied pressure of 138 kPa at a temperature of 90 ${}^{\circ }$C during 8 s. In a first step, the adhesive sheet is thermally activated for attachment to the Pure Copper Taffeta electrotextile. Next, the carrier sheet is removed, leaving behind the glue attached to the electrotextile. Later on, in a final step, the adhesive is activated once more to glue the electrotextile to the substrate.

### 3.2. Laser-Cutting

**Figure 3:**
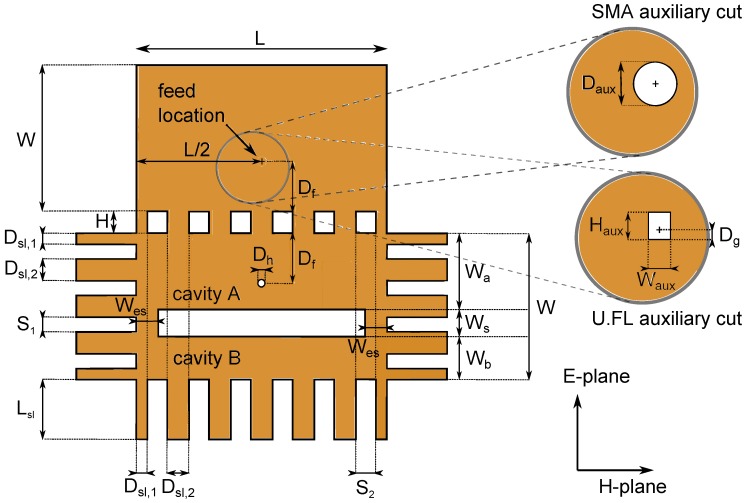
Optimized dimensions of the laser cut shape: H=4 mm, W=26.84 mm, L=45.94 mm, $W_{a}=16.49$ mm, $W_{b}=10.35$ mm, $W_{\mathrm{es}}=4$ mm, $W_{s}=5$ mm, $D_{f}=9.22$ mm, $D_{h}=0.65$ mm, $D_{\mathrm{aux}}=5$ mm, $H_{\mathrm{aux}}=2.85$ mm, $W_{\mathrm{aux}}=1.94$ mm, $D_{g}=0.9$ mm, $L_{\mathrm{sl}}=11$ mm, $D_{\mathrm{sl},1}=2$ mm, $D_{\mathrm{sl},2}=4$ mm, $S_{1}=2.71$ mm, $S_{2}=4$ mm.

Patterning fabrics through manual cutting offers insufficient accuracy to yield reproducible antennas. In addition, this approach is too labor-intensive and not suitable for mass production. Furthermore, scalpel cutting induces a significant risk of leaving loose filaments of the fabric, which is especially harmful to antenna performance when the apertures provided for the connector contain electrotextile frays, or when implementing slots which may be short-circuited by these frays. The use of an automatic laser cutter provides computerized sub-millimeter accuracy and not only prevents the cut edges from fraying, but also avoids misalignment between the slot and ground planes. A Pure Copper Taffeta patch, now glued to the adhesive on one side, is laser cut by a BRM90130 100 W CO${}_{2}$ laser cutter (BRM Lasers, 7102 DW Winterswijk, the Netherlands, online: https://brmlasers.eu/) in the shape shown in Figure 3 with a laser power of 25 W, at a speed of 100 mm/s, as it melts easier than the substrate, which is cut with a laser power of 80 W at a speed of 20 mm/s.

**Figure 5:**
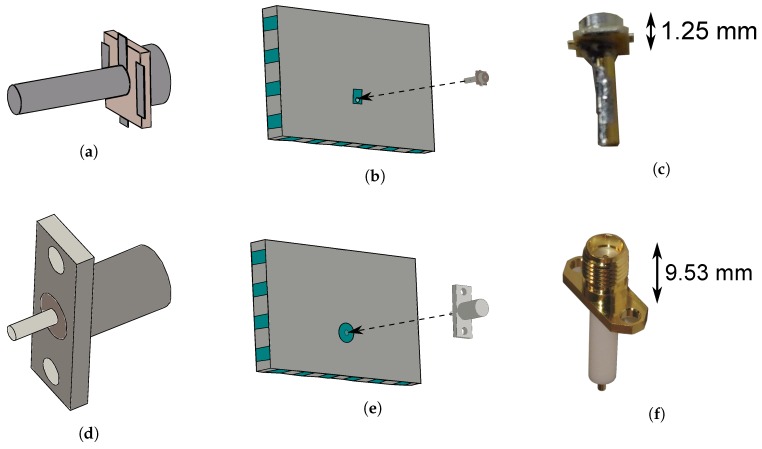
Final step of the fabrication process: Connector placement. (**a**) Model of U.FL connector with mounted pin, bottom side; (**b**) Already perforated cavity, insertion of U.FL connector with mounted pin into cavity; (**c**) U.FL connector with soldered pin; (**d**) Model of SMA connector with trimmed pin, bottom side; (**e**) Already perforated cavity, insertion of SMA connector with mounted pin into cavity; (**f**) SMA connector.

Depending on the applied connector, a different aperture is cut out for the via connection on the back plane (see Figure 3), above which the applied connector is placed. In order to accommodate an SMA connector, a cylindrical hole with a diameter of 5 mm is cut out. For a U.FL connector, a 1.94 mm to 2.85 mm rectangular hole is chosen instead. The U.FL connector is placed such that the connector’s side pin, which is connected to the connector’s center conductor (as shown further on the top part of Figure 5a), remains separated from the back plane of the antenna cavity.

Making use of the textile slabs shown in Figure 3 avoids the use of bulky and rigid tubelets as in [9,10,19]. Using electrotextile slabs instead of tubelets is also a necessity to achieve a low weight, as one single tubelet has a mass of 0.3 g and antennas for comparable operating frequencies easily require more than fifteen such tubelets [8,9,12]. Furthermore, the use of tubelets may cause ripping of the electrotextile and may hamper the integration possibilities of the antenna system. Textile slabs also avoid substrate compression due to tubelet punching [10]. Moreover, they guarantee that the sheet resistivity of the vertical walls is known in an exact and reproducible way. In contrast, vertical walls manufactured through embroidery exhibit a sheet resistance that is significantly higher than 0.05 Ω/□, as provided by the electrotextile applied in this work [22]. In addition, embroidered wires may also cause substrate compression [13,23].

### 3.3. Alignment Procedure

**Figure 4:**
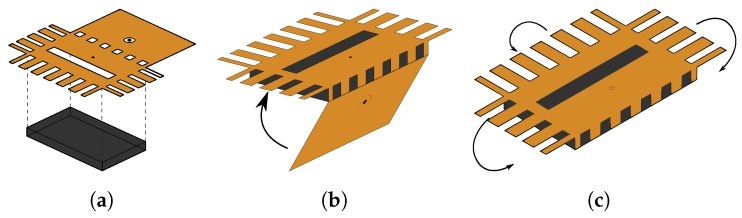
Antenna alignment procedure: steps 1 to 3. (**a**) Attachment of top (antenna slot) plane to substrate; (**b**) Folding around substrate; (**c**) Folding electrotextile around substrate and attachment of slabs.

The location of the feed $D_{f}$ and the dimensions of the connector slot (see $H_{\mathrm{aux}}$ and $W_{\mathrm{aux}}$ in the enlargement for U.FL and $D_{\mathrm{aux}}$ in the enlargement for SMA in Figure 3) for antenna feed placement are important design parameters. Therefore, it is imperative that a robust and straightforward alignment technique is used. Since the electrotextile patch is laser cut in one single piece, potential alignment errors are significantly more visible and thus less likely to happen compared to alignment procedures previously described in literature [6]. Furthermore, the novel workflow increases the ease of fabrication. As shown schematically in Figure 4a, to fix the antenna’s slot plane (coming from Section 3.2) to the substrate, the slot plane part of the electrotextile is thermally activated and connected to one side of the substrate, making use of the adhesive sheet connected to the electrotextile as described in Section 3.1. In the next step, the back plane is carefully folded around the substrate (Figure 4b), such that it precisely fits the back plane of the substrate. In Figure 4c, the electrotextile ribbons protruding the slot plane are folded around the substrate by a hot surface, thereby activating the adhesive and gluing the ribbons to the back plane.

### 3.4. Connector Placement

**Figure 6:**
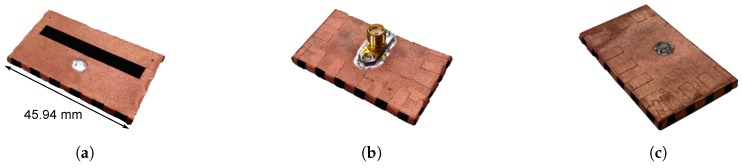
Final design. (**a**) Final design: slot plane; (**b**) Final design: back plane with SMA connector; (**c**) Final design: back plane with U.FL connector.

In the final step, the connector is placed. A U.FL connector requires an additional production step. Here, the 1-mm-diameter brass-gold pin is soldered to the inner conductor at the bottom side of the U.FL connector (see Figure 5a,c). This additional production step is unnecessary for SMA connectors, as they already possess an extruding pin as their center conductor. Instead, the dielectric around the SMA connector’s extruding pin is removed, as visible in Figure 5d. Next, the connector is positioned onto the prototype. The pin of the connector is trimmed to an appropriate size, after which it is punched through the substrate, which has been pierced previously. When the connector is in place, its ground pads are soldered to the back plane, after which the other end of the connector pin (which is now slightly protruding from the hole in the slot plane) is soldered to the slot plane, resulting in the final prototypes shown in Figure 6.

## 4. Simulation and Optimization

CST Microwave studio has been used for computer-aided electromagnetic simulation and optimization of the dimensions of the antenna. This has been done by performing a parametric analysis, allowing the design goals to be fulfilled. By optimizing the cavity widths $W_{a}$ and $W_{b}$, the locations of the resonance frequencies can be placed such that the antenna achieves a sufficiently large bandwidth. The length of the cavity L and the placement of the feed $D_{f}$ are selected for optimal impedance matching. Moreover, the length of the nonresonant slot and, hence, the dimensions of the additional electrotextile strips $W_{\mathrm{es}}$, fixing the actual slot length to $L-2W_{\mathrm{es}}$, (highlighted in step 3 of Figure 1) play the role of an additional parameter for tuning the coupling between both half mode cavities. All the aforementioned parameters are optimized to reach an optimal impedance match, while still fulfilling the predetermined impedance bandwidth and radiation goals. The optimized parameter values can be found in Figure 3. The widths $D_{\mathrm{sl},1}$ and $D_{\mathrm{sl},2}$ of the electrotextile slabs are chosen to be sufficiently large, whereas the optimized spacings $S_{1}$ and $S_{2}$ in between these slabs are small enough such that the fields are contained within the cavity, and lateral radiation losses become negligible. In the meantime, this choice of parameters keeps the antenna cavity mechanically flexible enough to satisfy the wearability constraints.

**Figure 7:**
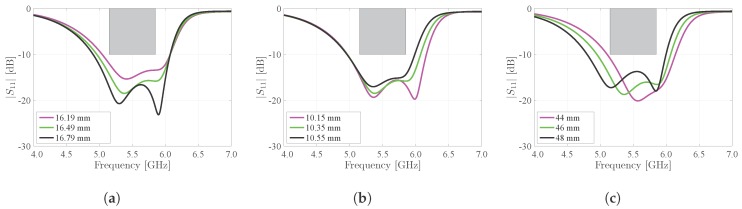
Simulated influence of cavity dimensions on the magnitude of the reflection coefficient $|S_{1,1}|$ of the antenna. (**a**) Influence of $W_{a}$; (**b**) Influence of $W_{b}$; (**c**) Influence of L.

In Figure 7, we demonstrate the effect of the main dimensions of cavity A ($W_{a}$ and L) and cavity B ($W_{b}$ and L) on the antenna’s reflection coefficient. Two distinct resonance frequencies can be observed, of which the lowest resonance frequency is related to cavity A and the highest resonance frequency is related to cavity B. Note that the resonance frequency of a cavity is inversely proportional to its dimensions [24]. The design requirements dictate that at least 90% of the maximal power available at the generator should be injected into the antenna over the complete frequency band of operation. This is indicated by the grey rectangular exclusion zone shown in the different subfigures, requiring that the power reflected by the antenna remains below 10%, or −10 dB, between 5.15 GHz and 5.85 GHz. To satisfy these requirements, both resonance frequencies should be carefully selected. Figure 7a,b show the influence of the half-mode cavity widths $W_{a}$ and $W_{b}$ on the resonance frequencies of the antenna, respectively. Since $W_{a}$ only affects the size of cavity A, by increasing $W_{a}$, the lowest resonance frequency decreases while the highest resonance frequency remains unaltered (Figure 7a). A similar behavior can be seen in Figure 7b, where an increase of $W_{b}$ leads to a downward shift of the highest resonance frequency, while the lowest resonance frequency remains constant. Also, Figure 7c shows that increasing the cavity length L lowers the frequency of operation of the antenna. Indeed, increasing L causes both cavities A and B to increase in size, thereby shifting their respective resonance frequencies downwards. However, for all considered variations on $W_{a}$, $W_{b}$ and L, the design requirements remain satisfied, proving the resilience of the antenna design with respect to fabrication tolerances.

**Figure 8:**
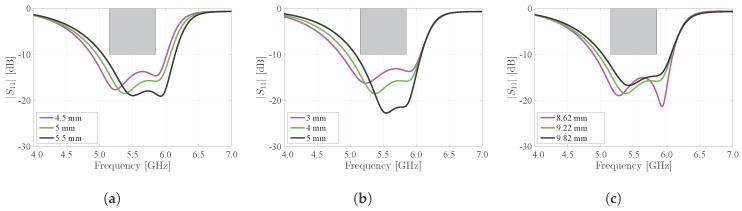
Influence of coupling and feed position on the magnitude of the reflection coefficient $|S_{1,1}|$ of the antenna. (**a**) Influence of slot width $W_{s}$; (**b**) Influence of electrotextile strip width $W_{\mathrm{es}}$; (**c**) Influence of feed position $D_{f}$.

In Figure 8, the influences of the nonresonant slot and the feed placement are demonstrated. Decreasing the spacing $W_{s}$ between both half-mode cavities increases the degree by which they are coupled. As such, the mode bifurcation effect is amplified. This is clear from Figure 8a, where the cavity resonances move further apart indeed. Moreover, it can be noted that decreasing $W_{s}$ while keeping the total antenna width W constant causes both half-mode cavities to become larger, which lowers their resonance frequencies. This explains the frequency shift visible on Figure 8a. Figure 8b,c show the influence of $W_{\mathrm{es}}$ and $D_{f}$, respectively, on the matching of the antenna. Hereby, a trade-off is made between keeping sufficient margins in terms of bandwidth, on the one hand, and maintaining a sufficient impedance match, on the other hand. Figure 8 proves that none of its three discussed dimensions are critical, as the margins on the deviations of the discussed parameters are sufficiently large. The same conclusions were made for Figure 7.

## 5. Experimental Results

This section discusses the measured figures of merit of the antenna prototypes in free space, as well as when the prototypes are subject to bending in a free space environment. Furthermore, the influence of humidity content and the proximity of the human body is measured.

### 5.1. Antenna Validation

#### 5.1.1. Figures of Merit in Free Space

**Figure 9:**
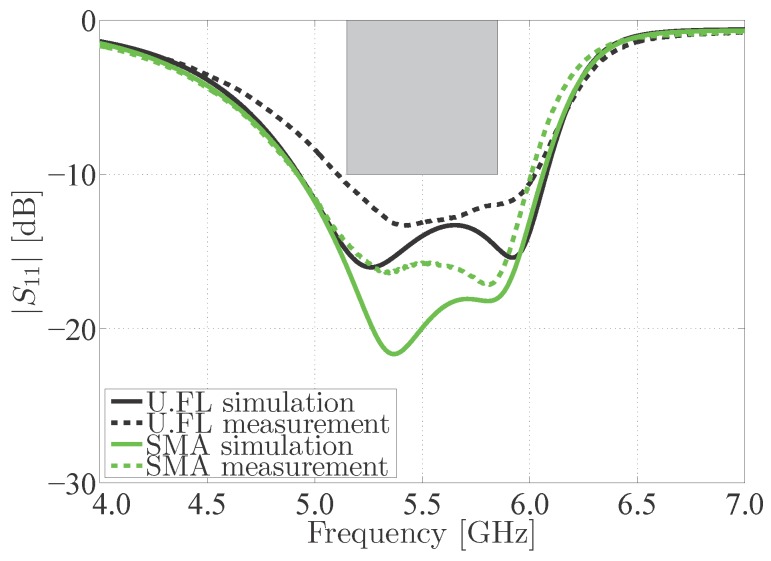
Magnitude of the reflection coefficient $|S_{1,1}|$ of the measured antennas in free space, with respect to a 50 Ω reference impedance.

The reflection loss and radiation performance of the proposed antenna have been measured in a full-anechoic chamber using an Agilent N5242A PNA-X Microwave Network Analyzer (Agilent Technologies, Santa Clara, CA 95051, USA, online: https://www.agilent.com/) [25] and an Orbit/FR DBDR antenna positioning system (MVG, 75005 Paris, France, online: http://www.orbitfr.com/). As seen in Figure 9, both the SMA and U.FL antennas clearly exhibit two separate resonances, whereby those of the antenna with SMA connector correspond best to the resonance frequencies as predicted by simulations. It is seen that the broadband design of the measured antenna indeed provides complete coverage of the desired frequency band. Moreover, the bandwidth of the SMA antenna is very similar to the simulated bandwidth. The model using the U.FL connector exhibits a reduced bandwidth as a result of an upwards shifted lower resonance frequency. This is the result of fabrication tolerances. Still, the U.FL prototype covers the desired frequency band. The prototypes of the U.FL antenna and the SMA antenna exhibit a −10 dB bandwidth of 920 MHz and 1.09 GHz, respectively, which categorizes them as UWB antennas [26]. In general, the small differences between measured and simulated results in Figure 9 are due to production tolerances as well as variations in material properties and in the environmental deployment conditions. Although laser patterning and the simplified and improved alignment procedure yield a significantly improved accuracy over hand cutting [27] and alignment, some small manufacturing and alignment errors remain that affect antenna performance. Moreover, the substrate and electrotextile’s material properties will slightly vary from batch to batch and with changing environmental conditions [27,28], giving rise to slightly different antenna characteristics.

**Figure 10:**
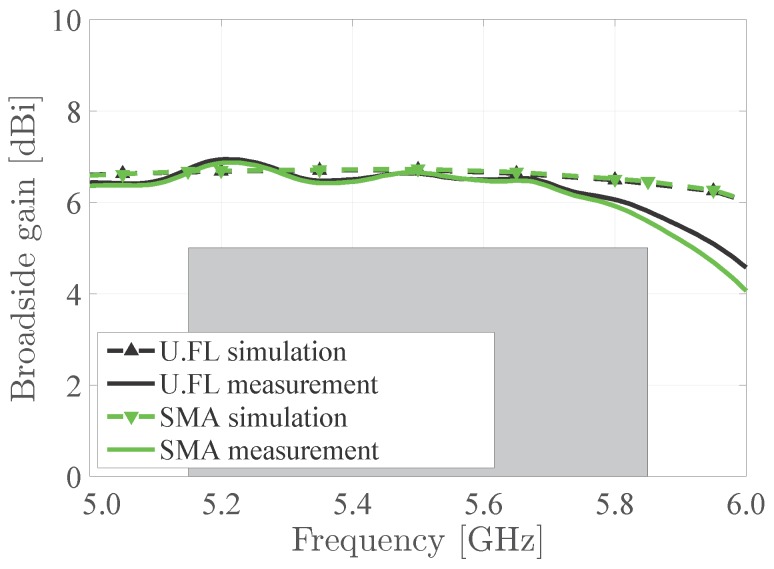
Comparison between simulated and measured gain of the measured antennas in free space.

The measured antennas maintain a stable gain over the desired frequency band. However, the antenna gain slightly drops off for higher frequencies, as seen in Figure 10. This is due to an increase of the substrate’s dielectric losses and the electrotextile’s conductor losses. This results in a lower radiation efficiency (see Table 1) and, hence, smaller antenna gain at higher frequencies. Yet, the measured broadside gain remains larger than 5 dBi over the entire frequency band under study. Furthermore, there is a good correspondence between measurements and simulations. Also, it is to be noted that the impact of the chosen connector remains small.

**Figure 11:**
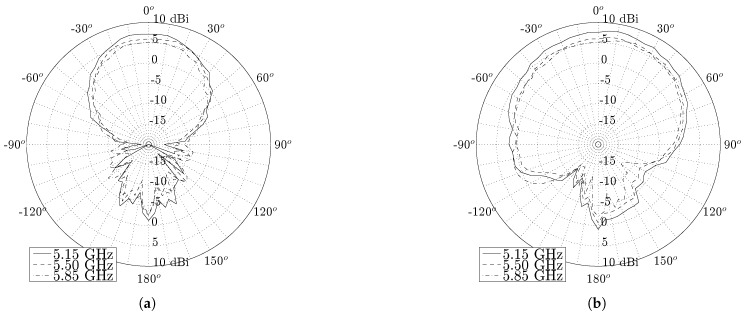
Free space radiation pattern of the U.FL antenna. (**a**) H-plane; (**b**) E-plane.

**Figure 12:**
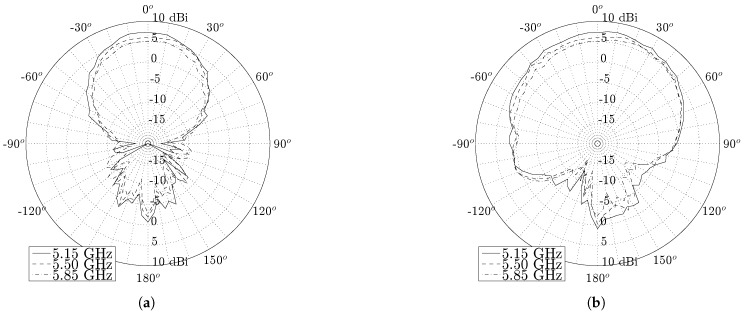
Free space radiation pattern of the SMA antenna. (**a**) H-plane; (**b**) E-plane.

**Figure 13:**
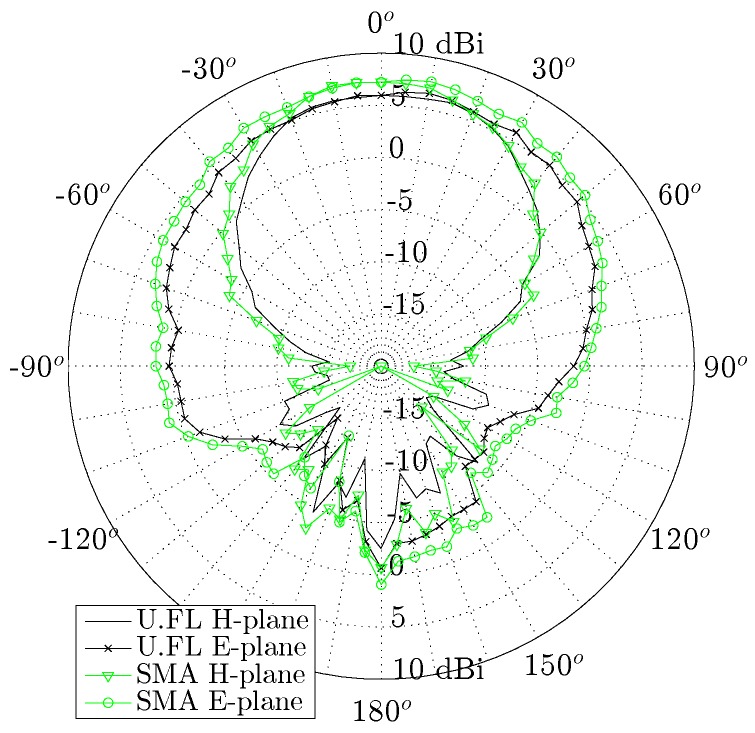
Measured radiation patterns of the U.FL antenna and the SMA antenna.

Figure 11 and Figure 12 show the radiation patterns in the H-plane and E-plane of the U.FL antenna and the SMA antenna, respectively. As predicted by simulations, both antennas exhibit very similar radiation patterns (see Figure 13) that remain stable over the desired band. The antenna efficiency, gain and front-to-back ratio of both antennas are evaluated at 5.15 GHz, 5.50 GHz and 5.85 GHz, as summarized in Table 1. Both antennas exhibit an efficiency higher than 70% and a gain above 5 dBi. It is thus proven that the antenna meets the free-space design goals formulated in Section 2.1.1.

#### 5.1.2. Figures of Merit on Body

**Figure 14:**
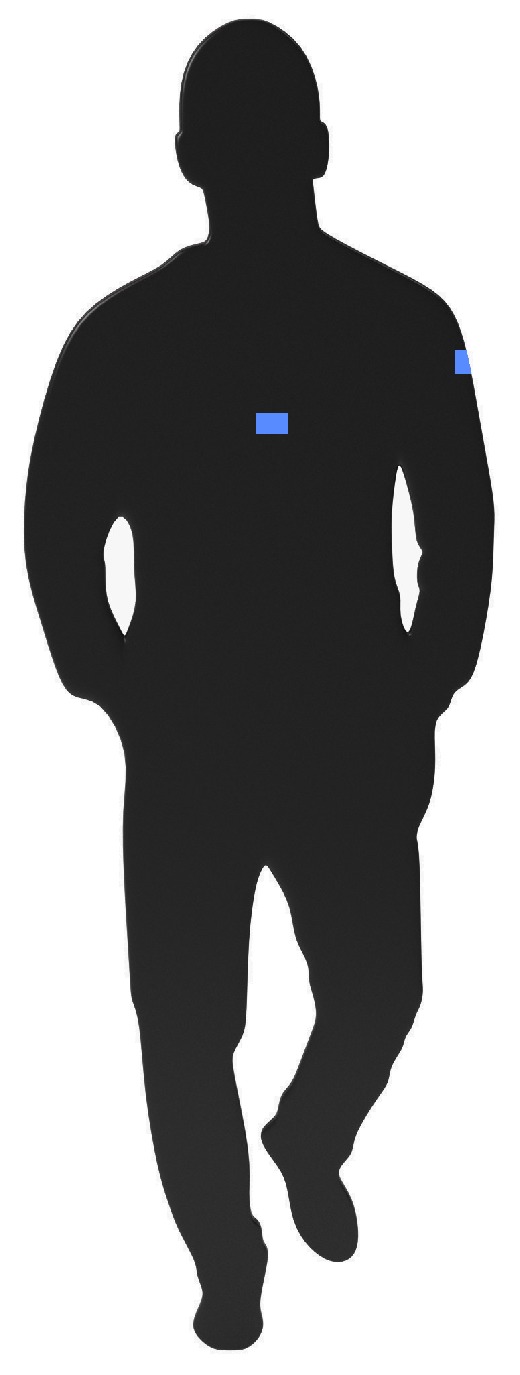
Measured on body placements of the antenna.

**Figure 15:**
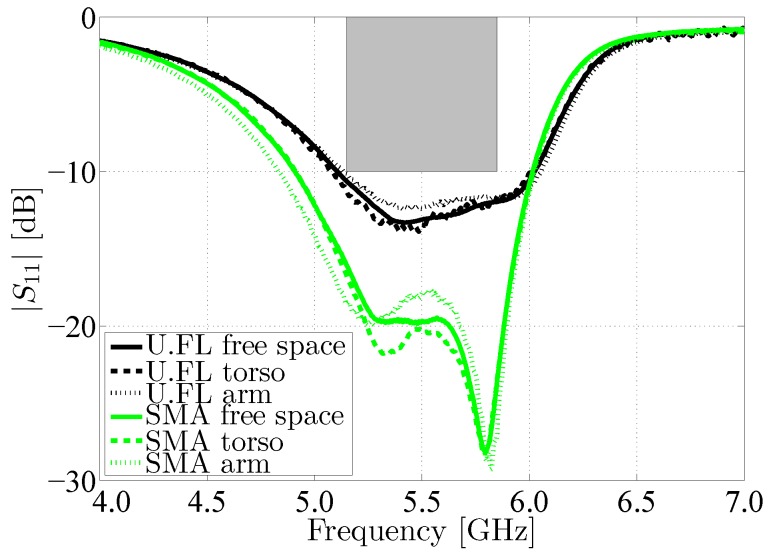
Magnitude of the reflection coefficient $|S_{1,1}|$ of the measured antennas in free space compared to measurement on torso and on the left arm, again with respect to a 50 Ω reference impedance.

Besides free-space measurements, the antenna performance was also evaluated in an on-body deployment scenario. Therefore, the antenna has been measured while worn on the torso of an average adult person of size 1.90 m and weight: 85 kg, as well as on the upper left arm of the test subject (see Figure 14). Figure 15 shows a very good antenna performance for all measurements, whether the antenna was characterized in free space, on torso or on the upper left arm. In all measured scenarios, the desired frequency band remains covered.

**Figure 16:**
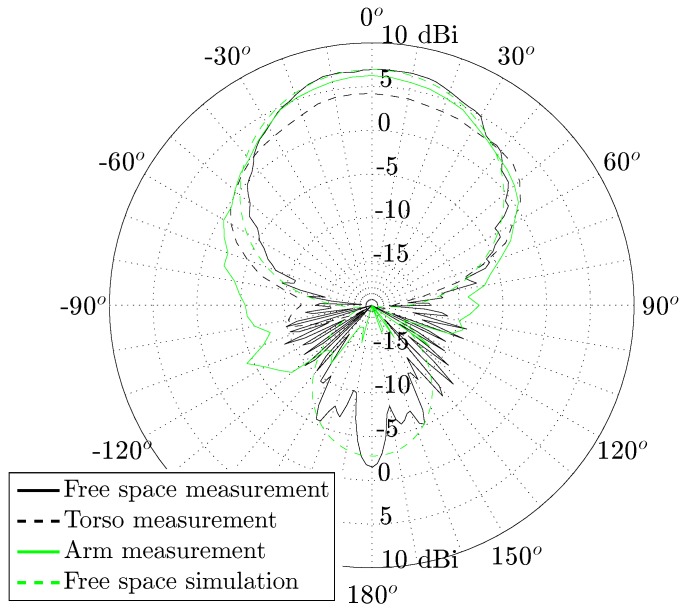
Measured vs. simulated 5.50 GHz H-plane radiation patterns of the U.FL antenna, free space vs. on body.

Figure 16 shows the radiation patterns of all aforementioned scenarios. Note the good correspondence with the simulated free space radiation pattern. Nevertheless, it is important to notice that the broadside gain reduces to some degree and the 3 dB-beamwidth slightly enlarges when deploying the antenna on the human torso. This effect can be attributed to the wider ground plane formed by the presence of the human body [29]. Yet, both measurements in which the antenna is placed on torso and on the upper left arm, exhibit a broadside gain larger than 4 dBi and a 3 dB-beamwidth larger than 60${}^{\circ }$. Observe that, when the antenna is deployed on the body, most of its backward radiation is suppressed due to body shadowing. Most electromagnetic fields radiated by the antenna towards the body are absorbed and dissipated as heat in the lossy body tissues. This effect also reduces the antenna’s radiation efficiency. Note that, thanks to the high antenna-body isolation of the adopted antenna topology, the reduction in radiation efficiency and gain remains small when deploying the antenna on body.

#### 5.1.3. Effects of Humidity Content

**Figure 17:**
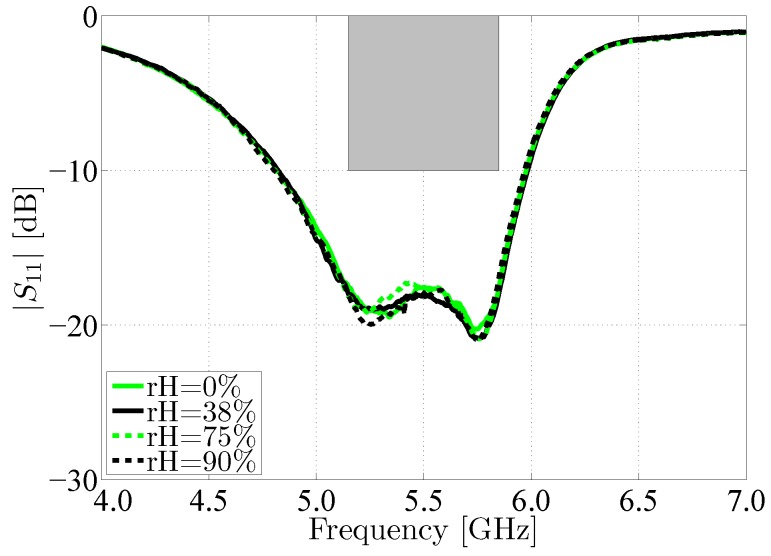
Magnitude of the reflection coefficient of the measured antenna for different relative humidities, with respect to a 50 Ω reference impedance.

The influence of varying relative humidity conditions on the performance of the antenna has also been investigated. Therefore, between every measurement, the antenna has been exposed to a specified relative air humidity in a climate test cabinet (WK 350 from Weiss Technik) for at least 16 h. The relative humidity of 0% was achieved by placing the antenna in a closet filled with a constant nitrogen gas flow, as this gas has a relative permittivity that is identical to that of dry air, but it contains no water vapor. Figure 17 shows that the humidity content of the antenna has little influence on antenna behavior. Under all measured circumstances, the antenna bandwidth and impedance matching remain stable. Given the low moisture regain of the selected materials, this is an expected result.

### 5.2. Effects of Antenna Deformation

**Figure 18:**
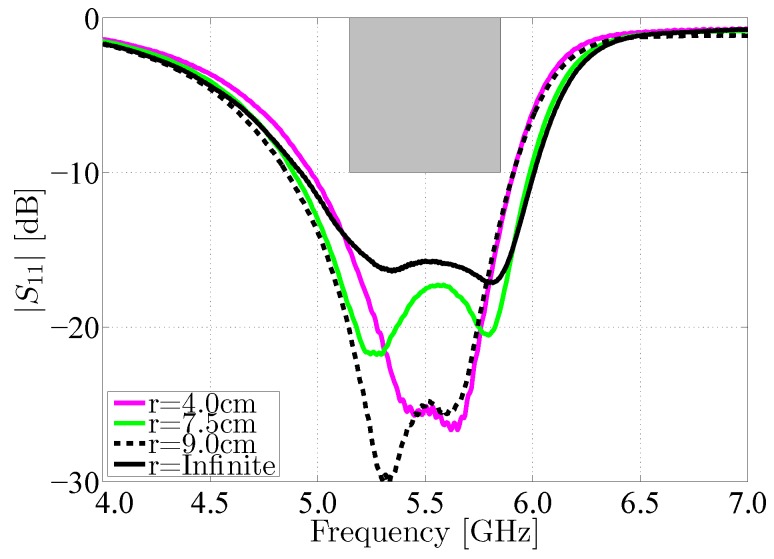
Magnitude of the reflection coefficient $|S_{1,1}|$ of the SMA antenna in free space (r = Infinite) compared to the magnitude of the reflection coefficient $|S_{1,1}|$ of the same antenna subject to bending around the X-axis, under different curvature radii r.

In a last set of tests, we have evaluated the effect of mechanical bending on the SMA antenna. Here, the antenna has been subjected to a selection of bending radii common to deployment at various locations on the human body. The magnitude of the reflection coefficient $|S_{1,1}|$ of the antenna has been investigated when bending the antenna around its X-axis. Figure 18 shows that under each of these circumstances, the antenna still covers the desired frequency band in terms of impedance match. The small variations in the antenna’s reflection coefficient are due to changes in antenna geometry, which may also result in substrate compression and a subsequent increase in substrate permittivity, as demonstrated in [30].

**Figure 19:**
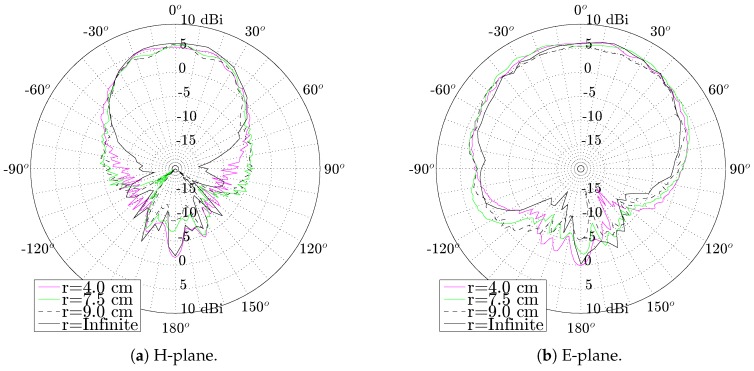
Measured 5.50 GHz free space radiation pattern of the SMA antenna bent around the X-axis, under different curvature radii r.

Both H-plane and E-plane radiations pattern of the antenna still meet the design goals when the antenna is bent. It can be observed from Figure 19 that both the −3 dB-beamwidth of the antenna and its antenna gain remain unaffected when the antenna is subjected to the investigated bending conditions.

## 6. Conclusions

A novel production process for wearable high-performance substrate integrated waveguide (SIW) cavity-backed textile antennas was proposed. The production process has been applied to fabricate two novel wearable and flexible cavity-backed SIW antennas for ultra-wideband operation in the [5.15–5.85] GHz band. To construct these antennas, materials commonly found in firefighter suits have been used. The resulting antenna prototypes benefit from an increased mechanical flexibility, while they maintain their radiation and impedance matching properties over the entire investigated band. Furthermore, the antenna is produced more accurately and requires fewer production steps than previous production methods, thereby resulting in an easier and faster fabrication procedure. The process creates smaller, lighter and mechanically more flexible antennas than cavity-backed SIW antennas fabricated using tubelets. Moreover, it produces more efficient antennas with more precisely known sheet resistivities than similar antennas produced by embroidery. This process may result in the production of actual unobtrusive and discretely wearable textile antenna systems on an industrial scale. Measurements show that the realized antennas continue to meet the predetermined specifications within the entire [5.15–5.85] GHz band, when they are subject to mechanical bending as well as when they are exposed to varying relative humidity conditions. This proves that the new manufacturing procedure allows for an accurate and swift way to realize robust, wearable high-performance antennas that are suitable as a wireless node in on-body communication systems.

## Figures and Tables

**Table 1:** Radiation efficiency, broadside gain and front-to-back ratio (FTBR) of the measured antennas.

Antenna Connector	U.FL			SMA		
Frequency [GHz]	5.15	5.50	5.85	5.15	5.50	5.85
Simulated total radiation efficiency [%]	91	89	82	90	92	87
Measured total radiation efficiency [%]	94	90	74	92	90	70
Simulated maximum gain [dBi]	6.72	6.83	6.62	6.71	6.79	6.60
Measured maximum gain [dBi]	6.73	6.64	5.81	6.65	6.66	5.59
Simulated FTBR [dB]	10.47	11.99	14.38	10.09	11.65	13.69
Measured FTBR [dB]	8.39	8.50	7.85	7.90	7.97	7.93
